# Mixed infections of fungal trunk pathogens and induced systemic phenolic compound production in grapevines

**DOI:** 10.3389/ffunb.2022.1001143

**Published:** 2022-09-15

**Authors:** Christopher M. Wallis, Zachary Gorman, Erin R. -A. Galarneau, Kendra Baumgartner

**Affiliations:** ^1^ Crop Diseases, Pest and Genetics Research Unit, San Joaquin Valley Agricultural Sciences Center, United States Department of Agriculture (USDA)-Agricultural Research Service, Parlier, CA, United States; ^2^ Plant Genetics Resources Unit, USDA-Agricultural Research Service, Geneva, NY, United States; ^3^ Crops Pathology and Genetics Research Unit, USDA-Agricultural Research Service, Davis, CA, United States

**Keywords:** Induced defence response, phenolics, Plant host resistance, grapevine, Diplodia seriata, Neofusicoccum parvum, Phaeomoniella chlamydospora

## Abstract

As grapevines mature in California vineyards they accumulate chronic wood infections by the Ascomycete fungi that cause trunk diseases, including Botryosphaeria dieback (caused by *Diplodia seriata* and *Neofusicoccum parvum*) and Esca (caused by *Phaeomoniella chlamydospora*). It is thought that such mixed infections become localized to separate internal lesions/cankers of the permanent, woody structure of an individual vine, but nonetheless the fungi all colonize the same vascular system. In response to infection by one pathogen, the host may initiate systemic biochemical changes, which in turn may affect the extent of subsequent infections by other pathogens. To test this hypothesis, we measured changes in phenolic compounds in the wood and lesion lengths of the pathogens, during sequential co-inoculations with different or identical pair-wise sequences of infection by *D. seriata*, *N. parvum*, or *P. chlamydospora*. Prior fungal infections only affected the development of subsequent *D. seriata* infections. Effects of fungal infections on phenolic compounds were variable, yet initial infection by *D. seriata* was associated with significantly higher concentrations of most phenolic compounds distally, compared to all other initial inoculation treatments. It was hypothesized that pre-existing phenolic levels can slow initial lesion development of fungal trunk pathogens, especially for *D. seriata*, but over time the pathogens appeared to overcome or neutralize phenolic compounds and grow unimpeded. These results demonstrate that effects of one fungal trunk pathogen infection is generally unable to distally affect another long-term, albeit shifts in host phenolics and other plant defenses do occur.

## Introduction

Grapevine (*Vitis vinifera* L.) fungal trunk pathogens are cosmopolitan and become widespread with high incidence in older vineyards (Bertsch et al., 2013). Although usually not fatal, the buildup of canker caused by fungi gradually results in yield reductions and, in combination with the build-up of nematode and viral pathogens in an orchard, often necessitate the entire replanting of the vineyard every 12 to 15 years (Baumgartner et al., 2019).

Several fungal trunk pathogens belonging to Botryosphaeriaceae and Herpotrichiellaceae families causes grapevine trunk diseases worldwide (Crous and Gams, 2000; Bertsch et al., 2013; Wilcox et al., 2015; Gramaje et al., 2018). In this sense, *Diplodia seriata* De Not., *Neofusicoccum parvum* (Pennycook & Samuels) Crous, Slippers, & A.J.L. Phillips, and *Phaeomoniella chlamydospora* (W. Gams, Crous, M.J. Wingf. & Mugnai) Crous & W. Gams are the most important trunk pathogens causing grapevine trunk diseases including Botryosphaeria dieback and esca, which can vary in symptomology and severity in different grapevine cultivars in California and elsewhere (Úrbez-Torres and Gubler, 2009; Travadon et al., 2013; Elena and Luque, 2016; Kaplan et al., 2016). Botryosphaeria dieback, potentially caused by *D. seriata*, *N. parvum*, and other related fungi including from genera such as *Botryosphaeria*, *Diplodia*, *Dothiorella*, *Lasiodiplodia*, *Neofusicoccum*, *Neoscytalidium*, *Phaeobotryosphaeria*, and *Spencermartinsia* (Úrbez-Torres and Gubler, 2011; Pitt et al., 2013; Rolshausen et al., 2013; Pitt et al., 2015; Yang et al., 2017), often appears as a lack of shoot growth of infected spurs in the Spring, with notable necrosis of buds and xylem, shoot dieback, and characteristic wedge-shaped perennial cankers in wood (Úrbez-Torres et al., 2006; Úrbez-Torres et al., 2008; Gramaje et al., 2018). This is somewhat distinct from esca disease, which is potentially caused by *P. chlamydospora* and other fungi (Crous and Gams, 2000; Andolfi et al., 2009; Díaz and Latorre, 2014), which, as a trunk disease, causes a variety of symptoms including black spots in the internal wood surrounding by pink/brown discoloration, brown to black vascular streaking, or dry wood with a silver appearance (Landi et al., 2011; Díaz and Latorre, 2014; Gramaje et al., 2018). *P. chlamydospora* also can be associated with Petri disease (Crous and Gams, 2000; Eskalen et al., 2007; Gramaje and Armengol, 2011), that has symptoms including black/brown streaking in xylem tissues, which can be observed as brown streaking in longitudinal cross-sections (Rooney-Latham et al., 2005; Eskalen et al., 2007; Gramaje et al., 2018).

Based on *in vitro* growth, *N. parvum* grew the most robustly, followed by *D. seriata* and then *P. chlamydospora* (Wallis, 2021). *P. chlamydospora* also has been described as having more limited wood-degrading activities than other fungal pathogens (Del Rio et al., 2004; Valtaud et al., 2009). Yet, of these three pathogenic fungi, *P. chlamydospora* was the most aggressive based on lesion length growth over time, followed by *N. parvum* and *D. seriata* (Galarneau et al., 2021). However, it should be noted that lesion sizes were similar among all three pathogens two weeks after inoculation, but *P. chlamydospora* lesions grew more over the next ten weeks, whereas most of the other cankers did not further develop (Galarneau et al., 2021).

A common defense that woody plants mount is the production of compounds called phenolics, which include subclasses such as flavonoids, which are precursors of cell wall strengthening compounds called tannins, and stilbenoids, which are thought to have direct antibiotic activities (Dixon, 2001). Previously, these three fungal pathogens were shown to induce phenolic compounds immediately around growing lesions and in nearby tissues, with levels greatest two months after inoculation (Galarneau et al., 2021). Stilbenoids were of particular interest in providing resistance to *N. parvum* and *P. chlamydospora* (Martin et al., 2009; Amalfitano et al., 2011; Khattab et al., 2020). Yet, the role of phenolics on fungal trunk pathogens remains uncertain. Antifungal activity of the phenolic stilbenoids was determined *via in vitro* amended plate assays for fungal trunk pathogens (Lambert et al., 2012). Some evidence exists that fungicidal activity of phenolics *in planta* might be lacking for certain fungi such as *N. parvum*, but present for others such as *D. seriata* (Lambert et al., 2012; Galarneau et al., 2021).

The induction of phenolic compounds distal from the inoculation site has not been as thoroughly studied. Massonnet et al. (2017) explored systemic changes in the grapevine transcriptome in leaves and local wood due to *N*. *parvum* infection, but chemical analyses of phenolics was limited to the area of local inoculation. This experiment explicitly measured the induction of phenolic compounds in distal woody branches that were not directly inoculated with fungal trunk pathogens. Because fungal pathogens could change phenolic compound levels distally and phenolics could affect fungal growth (Galarneau et al., 2021), it was hypothesized that an initial fungal infection could impact subsequent fungal infections in different branches (Wallis et al., 2008). A total of 15 different combinations were tested, with plants left uninfected, mock-inoculated, or initially infected with *D. seriata*, *N. parvum*, or *P. chlamydospora* and subsequently followed by infection on a different branch by *D. seriata*, *N. parvum*, or *P. chlamydospora*. Results determined how fungal trunk pathogens altered phenolic levels distally from infection sites, and whether or not that affected development of an addition canker infection. Knowledge obtained has implications in integrated disease management approaches aimed at controlling fungal trunk diseases and should increase insights into the role of phenolics in plant-pathogen interactions.

## Materials and methods

**Figure 1:**
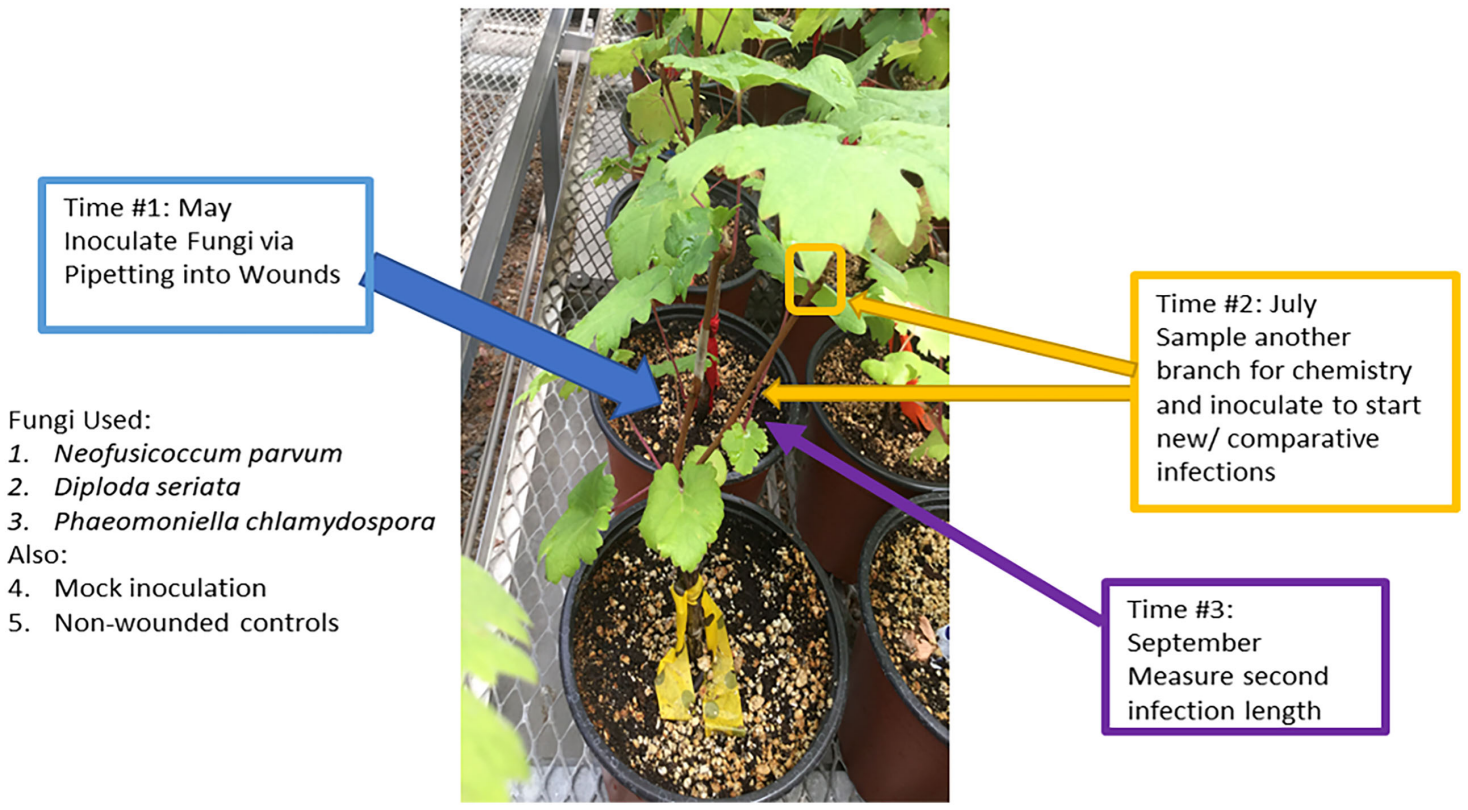
Overview of the experimental procedures. Second inoculations were applied on a second shoot two months after initial inoculation treatments, and at the same time woody shoots were sampled for chemistry.

A total of 90 ‘Cabernet Sauvignon’ grapevines on ‘101-14MG’ rootstocks obtained from a commercial nursery were used across all treatments in the experiment. In May 2019 (“Time 1”), six grapevines, each with at least two shoots, were assigned the combination of one of the following initial inoculation treatments and subsequent (second) inoculation treatments (**Figure 1**). The initial inoculation treatments were as follows: *D. seriata*, *N. parvum*, *P. chlamydospora*, non-inoculated – wounded control (NIW), or non-inoculated - non-wounded control (NINW). Inoculum consisted of suspensions of 1,000,000 mycelium fragments per mL for *D. seriata* and *N. parvum*, and 100,000 spores per mL for *P. chlamydospora* (made by using a homogenizer of each fungal species grown for one week in potato dextrose broth) (Travadon et al., 2013). A larger concentration of mycelia fragments was inoculated due to some mycelia fragments being dead or unviable because they were potentially too old/no-longer-growing fragments from culture plates or were destroyed in the homogenization process as estimated *via* microscopy (i.e. those appearing desiccated or damaged were roughly 10%); therefore, greater mycelia fragment inoculum would be needed to correspond with concentrations of *P. chlamydospora* conidia inoculum that should have greater viability (Wallis, 2021). A power drill was used to make a small wound at the inoculated site, which was located approximately 10 cm above the scion-rootstock grafting site on one shoot. Pathogen inoculum (100 µL) for inoculated shoots or sterile potato dextrose broth for NIW plants was pipetted into the wound and immediately covered with parafilm. NINW controls were left intact; there was no wound or inoculum (Travadon et al., 2013).

In July 2019 (“Time 2”), immediately prior to the second inoculations, a 10 to 15-cm-long segment of stem was collected from the apical end of a third shoot or the shoot about to receive the second inoculation (i.e., a different shoot from the one inoculated at “Time 1” in May 2019), in order to assess systemic (distal) changes in host chemistry away from the initial inoculation site. Stem segments were immediately flash-frozen in liquid nitrogen and stored in a -20°C freezer for further processing. For the second inoculation, plants were inoculated on a separate shoot with *D. seriata*, *N. parvum*, or *P. chlamydospora*, as described above. There were no NIW or NINW controls for the second inoculation.

In September 2019 (“Time 3”), stems inoculated in July 2019 (“Time 2”) were harvested to measure lesion lengths of the second inoculation. To measure the internal lesions, the bark above and below an inoculation site was removed and the brown or black discoloration of the stem tissue underneath was measured with a ruler. To confirm infection by attempting to re-isolate the fungi, 1-mm segments from both the apical and basal ends of the lesion (or at inoculation site, in the case of the NIW controls) were collected and plated on potato dextrose agar (PDA) plates following brief sterilization of the wood fragments for 30 seconds in 10% bleach. To confirm pathogen identity, DNA was extracted from approximately 25% of the resultant fungal colonies for PCR amplification of the ITS region (White et al., 1990), and ITS sequences were compared to those of the original isolates (Swofford, 1999).

The methods of Wallis et al. (2008) and Wallis and Chen (2012) were used to extract and analyze phenolic compounds. The methanol and other solvents used were obtained from Thermo-Fisher Scientific (Waltham, MA, USA) and standards were from Sigma-Aldrich (St. Louis, MO, USA). The collected stem tissues were pulverized with mortar and pestle in liquid nitrogen, and then 0.10 g of the tissue was placed into 1.5 mL centrifuge tubes. The tissue was then twice-extracted overnight at 4°C in 0.5 mL methanol, with a final extract volume of 1 mL obtained. High performance liquid chromatography (HPLC) then was conducted using a Shimadzu (Columbia, MD, USA) LC-20AD pump based liquid chromatograph equipped with Supelco Ascentis C18 (Sigma-Aldrich, St. Louis, MO, USA) column and a Shimadzu PDA-20 photodiode array detector. Each sample had 50 µL of the methanol extract injected. Compounds were identified by matching retention times with commercial standards or putatively identified *via* liquid chromatography-mass spectrometry using a Shimadzu LCMS2020 system running similar conditions to the original HPLC. Quantification was performed by using a standard curve of commercially available compounds from the same phenolic subclass, i.e., ferulic acid for hydroxycinnamic acid derivatives, procyanidin B2 for proanthocyanins, catechin for flava-3-ols, quercetin glucoside for flavonoid glycosides, and resveratrol for stilbenoids (Wallis and Chen, 2012; Wallis et al., 2020). This was used to convert peak areas into mg/g fresh weight.

Statistical analyses were performed using IBM (Armonk, NY, USA) SPSS statistics version 24 with α = 0.05. Spearman’s correlations were utilized to find associations between lesion lengths and phenolic levels (total phenolics or the sums of each individual phenolic subclass). Analyses of variance (ANOVAs) were used to compare lesion sizes or total phenolics (and the sums of phenolic subclasses) with initial inoculation treatment as the independent variables. Multivariate analysis of variance (MANOVA) was used to analyze the effects of the initial inoculation treatments on all individual phenolics as well, with follow-up ANOVAs and Least Significant Difference (LSD) tests performed when significant (*P* < 0.05).

## Results and discussion

**Figure 2:**
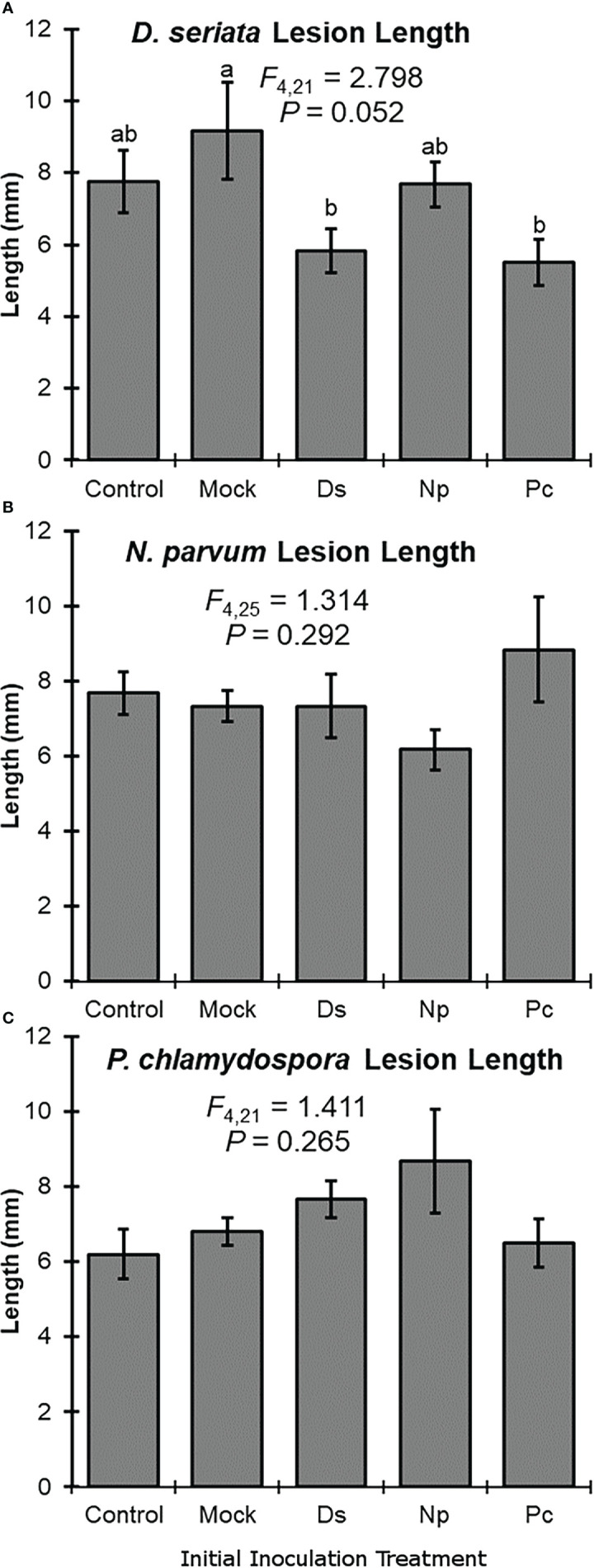
Mean lesion lengths (± SE) after the second inoculation with: **(A)** *Diplodia seriata*, **(B)** *Neofusicoccum parvum*, or **(C)** *Phaeomoniella chlamydospora*. Differences among initial inoculation treatments are noted with different letters (statistical differences by least significant differences tests). Initial inoculation treatments are as follows (on the X axis): Control, NINW; Mock, NIW; Ds, *Diplodia seriata*; Np, *Neofusicoccum parvum*; Pc, *Phaeomoniella chlamydospora*.

Initial inoculation treatment had a significant effect on lesion length after the second inoculation with *D. seriata*. Following initial inoculation with either *D. seriata* or *P. chlamydospora*, lesion lengths of *D. seriata* were significantly smaller, compared to plants with an initial inoculation treatment of wounding alone (i.e., the NIW controls); *D. seriata* lesions on plants with initial inoculation treatments of NINW or *N. parvum* were intermediate in length (**Figure 2**). Initial inoculation treatment had no effect on lesion length after the second inoculation with *N. parvum* or *P. chlamydospora* (**Figure 2**). In other words, neither *N. parvum* lesions nor *P. chlamydospora* lesions (of the second inoculation) varied significantly according to initial inoculation treatment.

**Figure 3:**
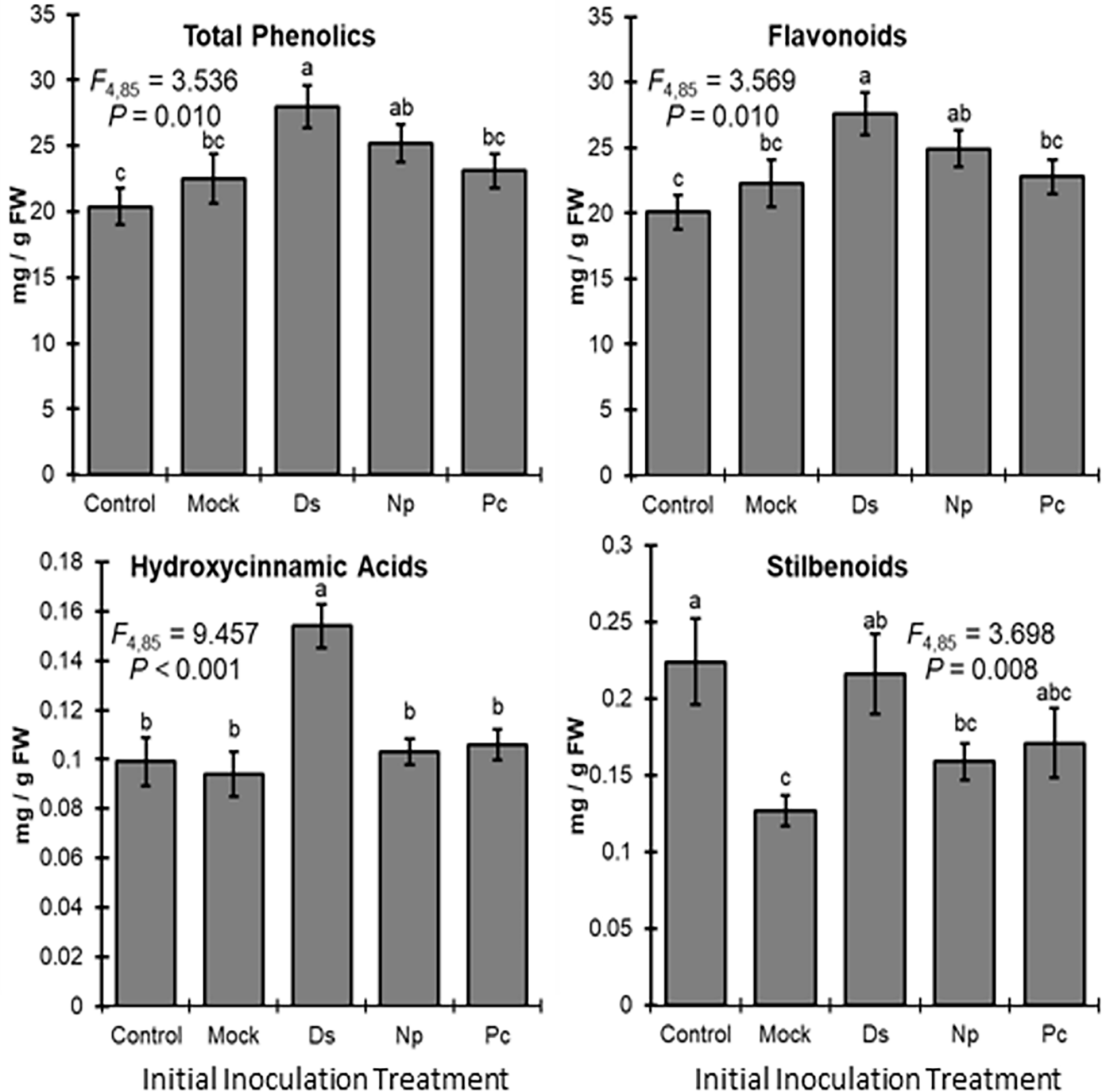
Mean phenolic compound levels (± SE) in the woody stem after the initial inoculation with: Control, NINW; Mock, NIW; Ds, *Diplodia seriata*; Np, *Neofusicoccum parvum*; Pc, *Phaeomoniella chlamydospora*. Differences among initial inoculation treatments are noted with different letters (statistical differences by least significant differences tests). Initial inoculation treatments are as follows (on the X axis): Control, NINW; Mock, NIW; Ds, *Diplodia seriata*; Np, *Neofusicoccum parvum*; Pc, *Phaeomoniella chlamydospora*.

**Figure 4:**
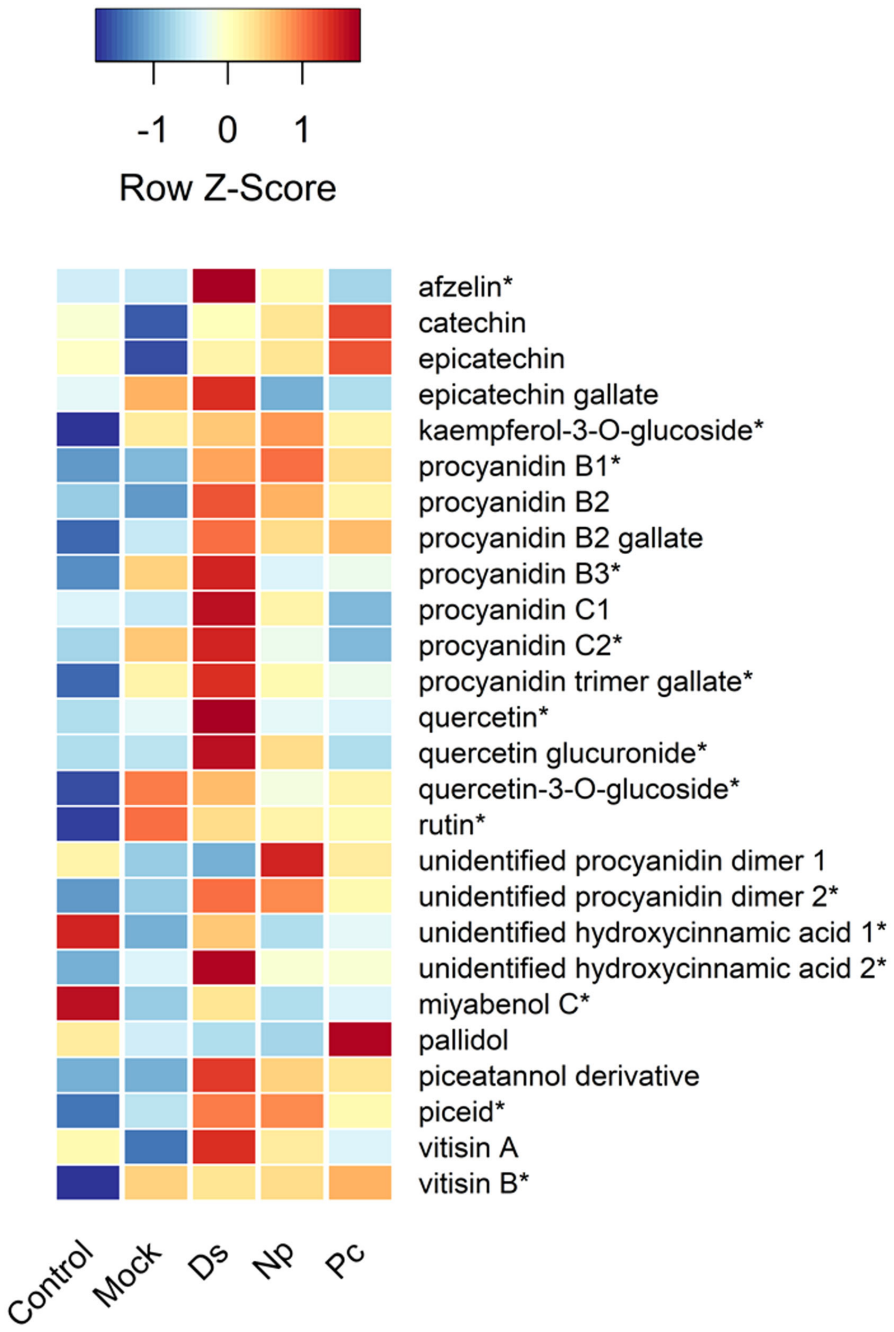
Heat map showing the relative amounts (Z-scores) of individual phenolic compounds quantified in this study and taken just prior to second inoculations. Control, NINW; Mock, NIW; Ds, *Diplodia seriata*; Np, *Neofusicoccum parvum*; Pc, *Phaeomoniella chlamydospora*. *Indicates significance at *P* < 0.05, with statistics provided in **Table 1**.

Phenolic levels in the shoots varied significantly, overall, depending on the initial inoculation treatment in a separate shoot. Total phenolic levels and total flavonoid levels were significantly greater in plants initially inoculated with *D. seriata*, compared to those initially inoculated with *P. chlamydospora* and both types of controls (NIW and NINW) (**Figure 3**). Total hydroxycinnamic acid derivatives were greater in plants initially inoculated with *D. seriata* compared to all other treatments (**Figure 3**). Total stilbenoid levels were greater in plants initially inoculated with *D. seriata* compared to plants initially inoculated with *N. parvum* and the NIW controls (**Figure 3**). Based on the results of MANOVA, there was a significant effect of the initial inoculation treatment on individual phenolic levels (Pillai’s trace = 2.332; *F* = 3.389; *P* < 0.001). Effects of the initial inoculation treatments were overall variable, but there was a trend for higher levels of individual phenolics in plants initially inoculated with *D. seriata* than the other treatments. Sixteen of twenty-six quantified phenolics were significant by individual follow-up ANOVAs (*P* < 0.05) (**Table 1**; **Figure 4**).

**Table 1:** Analysis of variance stats (*F-* and *P-*values) demonstrating compounds with differing means between treatments used in this study.

Compound Class	Compound	*F_4, 85_*	*P*
flavonoids	afzelin	5.975	0.000
	catechin	2.204	0.075
	epicatechin	1.783	0.140
	epicatechin gallate	1.967	0.107
	kaempferol-3-O-glucoside	10.935	0.000
	procyanidin B1	2.482	0.050
	procyanidin B2	0.684	0.605
	procyanidin B2 gallate	1.188	0.322
	procyanidin B3	3.008	0.023
	procyanidin C1	1.980	0.105
	procyanidin C2	3.417	0.012
	procyanidin trimer gallate	3.204	0.017
	quercetin	10.524	0.000
	quercetin glucuronide	3.259	0.015
	quercetin-3-O-glucoside	4.651	0.002
	rutin	9.440	0.000
	unidentified procyanidin dimer 1	0.559	0.693
	unidentified procyanidin dimer 2	3.761	0.007
hydroxycinnamic acids	unidentified hydroxycinnamic acid 1	3.905	0.006
	unidentified hydroxycinnamic acid 2	10.808	0.000
stilbenoids	miyabenol C	9.824	0.000
	pallidol	1.144	0.342
	piceatannol derivative	2.368	0.059
	piceid	3.840	0.006
	vitisin A	1.983	0.104
	vitisin B	4.997	0.001

See text and **Figure 4** for significant pairwise separations.

Two hydroxycinnamic acid derivatives (one with greater amounts in non-wounded controls that all other treatments except those infected with *D. seriata*, and the other greater in plants infected with *D. seriata* than all other treatments), procyanidin B1 (greater in plants infected with *D. seriata* and *N. parvum* than non-wounded or mock controls), procyanidin B3 (greater in plants inoculated with *D. seriata* than non-wounded controls), procyanidin C2 (greater in plants inoculated with *D. seriata* than all other plants except those mock-inoculated), a procyanidin C gallate (greater in plants infected with *D. seriata* than non-wounded controls and plants infected with *P. chlamydospora*), an unidentified procyanidin B (greater in plants infected by *D. seriata* or *N. parvum* than non-wounded controls), miyabenol C (greater in healthy controls than all other plants, and greater in *D. seriata* infected plants than those mock-inoculated or inoculated with *N. parvum*), piceid (greater in plants infected with *D. seriata* or *N. parvum* than healthy controls or mock-inoculated plants), vitisin B (lower in healthy controls than all other plants), afzelin (greater in plants infected with *D. seriata* than all other plants), kaempferol glucoside (lower in healthy controls than all other plants), quercetin (greater in *D. seriata* inoculated plants than all other plants), quercetin glucuronide (greater in *D. seriata* inoculated plants than all other plants except those infected with *N. parvum*), and quercetin glucoside (lower levels in healthy controls than all other plants). No significant correlations were observed between lesion lengths and total phenolic levels, including with total amounts of phenolic subclasses (in every case, *P* > 0.05).

Our findings suggest that *D. seriata* infection may initiate systemic biochemical changes, in terms of total phenolics and specific individual compounds, in the grape stem. Wounding alone had a slightly less significant effect on stem chemistry. These changes in host chemistry following initial inoculation treatment with *D. seriata* at least partially affected lesion development, especially for subsequent *D. seriata* infections. Interestingly, initial inoculation with *P. chlamydospora* was associated with smaller lesions after a second inoculation with *D. seriata*, even though the biochemical changes immediately before the second inoculation were relatively minor.

Although *D. seriata*, *N. parvum* and *P. chlamydospora* cause internal wood lesions, they cause different diseases in the vineyard with different symptoms with the former two fungi causing wedge-shaped cankers and the later mostly black streaking in vascular tissues (Martin and Cobos 2007; Amponsah et al., 2011; Garcia et al., 2021). Pathogenesis is different as well (Garcia et al., 2021). Indeed, both *D. seriata* and *N. parvum* were observed to trigger differential responses in grapevine calli exposed to their extracellular proteins, suggesting both might produce toxins, albeit in different amounts (Benard-Gellon et al., 2015). Benard-Gellon et al. (2015) and Stempien et al. (2018) also observed that *N. parvum* induced greater transcription of defense genes than *D. seriata*, although greater induction of defensive metabolites was not observed in this study, based on observed soluble phenolic levels. However, soluble phenolic levels could be influenced by more than host induction, including the possibility that the fungal pathogens may degrade them (Massonnet et al., 2018), or they could be incorporated into phenolic macromolecules that were not examined.

As for lesion lengths after second inoculation with *N. parvum* or *P. chlamydospora*, prior inoculation treatments had no effect. It was hypothesized that greater phenolic levels might reduce initial establishment of *N. parvum*, but that it could quickly adapt and overcome this inhibition and grow by rapidly catabolizing phenolic compounds, resulting in no significant correlations. This is supported by evidence that *N. parvum* not only possess genes that perform phenolic compound catabolism, but also can greatly remove phenolic compounds in cultures (Gluck-Thaler et al., 2018; Khattab et al., 2020). Indeed, *Neofusicoccum* spp. in general were discovered to have greater expansion of gene families such as secreted cell wall degrading enzymes, secondary metabolism, and transporters than *Diplodia* spp., which was concluded to result in greater lesion sizes and presumably greater capacity to overcome host defenses (Garcia et al., 2021). In other words, *N. parvum* likely is capable of both degrading phenolics and other defense compounds (Massonnet et al., 2018), as well as transporting host-derived antimicrobial compounds out of their cells (Denny et al., 1987).

Regarding *P. chlamydospora*, a previous study observed that, similar to *N. parvum*, *P. chlamydospora* might also have increased capacity to tolerate phenolic compounds (Galarneau et al., 2021). *P. chlamydospora* also appears to be unaffected by stilbenoid compounds *in vitro* (Lambert et al., 2012). That said, capacity of this fungal species to catabolize phenolics remains unclear. Taken together, the ability to overcome phenolic production likely resulted in our observations that there were no significant effects from prior infections on *P. chlamydospora* lesion sizes. However, further studies are needed to provide additional insights into the pathogenesis of *P. chlamydospora*, similar to those performed on *N. parvum* and *D. seriata*.

Results obtained in this study represent just one set time of 60 days for secondary infections, with phenolics only observed at that time. It was very likely that potential inductions of phenolics would be anticipated to vary over time, for instance, Galarneau et al. (2021) observed phenolic concentrations around fungal lesions continued to increase as late as 90 days and potentially beyond. Therefore, phenolics could be greater if a later timepoint was chosen, and, accordingly, impacts on developing secondary infections could be greater. Additional studies with greater timepoints, both earlier and later, are warranted but were not performed in these experiments due to limits in greenhouse space and available resources.

Likewise, the age of the plants should be considered in light of these results. Certain fungal trunk diseases appear to infect only older, established grapevines in vineyards, and infections of smaller, potted plants may have influenced results accordingly. Thus, repetition of this study in a vineyard at least seven-years-old could verify the relevance of these results to field plantings.

In conclusion, this study increases some insight into mixed infections, and how they are potentially mediated by phenolic compounds. Overall, greater efforts need to be made to examine how mixed infections affect each other and overall plant health, as such studies are generally lacking (Bonello et al., 2008; Dutt et al., 2021). A previous study by Bonello et al. (2008) found increased *Diplodia pinea* growth in pines that were previously infected with *Heterobasidion annosum*, and another observed increased, decreased, or no effects on secondary pathogen growth based on different combinations of pea-infecting fungal pathogens (Dutt et al., 2021). These works, together with this study, which observed decreased or no effects on pathogen growth depending on different combinations of pathogenic fungi, suggested that interactions between different pathogens on the same host are largely driven by the individual characteristics of the specific pathogens. These results further suggest that the capacity of different pathogens to tolerate, degrade, and/or efflux defensive host metabolites, as well as the ability to share host resources for development when in close proximity, are important factors in these multi-pathogen interactions.

Thus, examining the role of phenolics can be argued a good choice to begin to untangle how mixed infections affect one another and their shared host. That said, it is clear phenolics are not the only factor that drive lesion development, and that other mechanisms are likely to have impacted the observed lesion development in this study. Future studies are warranted to examine possible interactions between pathogens on grapevines that include observations of phenolics and many other defense-related factors such as PR-proteins, volatiles, and structural changes, among others.

Measurements of plant hormones or associated upregulated transcripts also could be assessed, including those involved with Systemic Acquired Resistance (SAR) (i.e., salicylates) and Induced Systemic Resistance (ISR) (i.e. jasmonates) as defensive pathways remain poorly understood in grapevines. However, it could be hypothesized that these fungal pathogens should generically trigger the SAR pathway due to a facultative necrotic-feeding lifestyle, albeit both SAR and ISR could induce phenolics as part of the defense responses they mount (Wallis and Galarneau, 2020).

## Data availability statement

The datasets presented in this study can be found in online repositories. The names of the repository/repositories and accession number(s) can be found below: https://doi.org/10.15482/USDA.ADC/1527769.

## Author contributions

CW conceived, obtained funding, executed, performed analyses and experiments, performed statistics, and wrote the manuscript for this study. ZG assisted with the statistics and writing of the manuscript. EG and KG assisted with the execution of the experiment. All authors contributed to the article and approved the submitted version.

## Funding

This work was funded *via* allocated fund to the USDA-Agricultural Research Service, Program #2034-22000-012-00D.

## Acknowledgments

The author thanks Mala To, Justin King, Ben Tanielian and Nalong Mekdara for their technical assistance in this work. Mention of trade names or commercial products in this publication is solely for the purpose of providing specific information and does not imply recommendation or endorsement by the U.S. Department of Agriculture. USDA is an equal opportunity provider and employer.

## Conflict of interest

The authors declare that the research was conducted in the absence of any commercial or financial relationships that could be construed as a potential conflict of interest.

## Publisher’s note

All claims expressed in this article are solely those of the authors and do not necessarily represent those of their affiliated organizations, or those of the publisher, the editors and the reviewers. Any product that may be evaluated in this article, or claim that may be made by its manufacturer, is not guaranteed or endorsed by the publisher.
